# Individual and community-level determinants of childhood vaccination in Ethiopia

**DOI:** 10.1186/s13690-021-00581-9

**Published:** 2021-04-20

**Authors:** Setegn Muche Fenta, Haile Mekonnen Fenta

**Affiliations:** 1 Department of Statistics, Faculty of Natural and Computational Sciences, Debre Tabor University, Debre Tabor, Ethiopia; 2 Department of Statistics, College of Science, Bahir Dar University, Bahir Dar, Ethiopia

**Keywords:** Child vaccination, Ordinal, Multilevel, Ethiopia

## Abstract

**Background:**

Vaccines are one of our most important tools for preventing outbreaks and keeping the world safe. Most unvaccinated children live in the poorest countries including Ethiopia. Therefore, this study aimed to identify the determinants of vaccination coverage among children aged12–23 months in Ethiopia.

**Methods:**

A cross-sectional secondary data were obtained from the 2016 Ethiopian Demographic and Health Survey data (EDHS). A total of 1929 children were included. A Multilevel Proportional Odds Model was used to identify the individual and community-level factors associated with child vaccination.

**Result:**

Among 1, 929 children, only 48.6% (95% CI: 46.3 to 50.8%) were fully vaccinated while 37.8% (95% CI: 35.7 to 40.1%) were partially vaccinated.. The multilevel ordinal logistic regression model reveled that housewife mother (AOR =1.522, 95%CI: 1.139, 2.034), institutional delivery (AOR =2.345, 95%CI: 1.766, 3.114),four or above antenatal care visits (AOR = 2.657; 95% CI: 1.906, 3.704), children of mothers with secondary or higher education (AOR = 2.008; 95% CI: 1.209, 3.334),Children whose fathers primary education (AOR = 1.596; 95% CI: 1.215, 2.096), from the rich households (AOR = 1.679; 95% CI: 1.233, 2.287) were significantly associated with childhood vaccination.

**Conclusion:**

Child vaccination coverage in Ethiopia remains low. Therefore, there is a need to increase child vaccination coverage by promoting institutional delivery and prenatal care visits, as well as maternal tetanus immunization. Besides, public initiatives needed to improve child vaccination coverage, women’s and husband’s education, poor women, and further advancement of health care services for poor women, housewife women, women living in remote areas should be made to maintain further improvements in child vaccination. Furthermore, policies and programs aimed at addressing cluster variations in child vaccination need to be formulated and their implementation must be strongly pursued.

## Background

In 2019, nearly 5.2 million children died, and about 14,000 children still die globally every day. About 80% of the 5.2 million child deaths have occurred in sub-Saharan Africa and Central and Southern Asia. Sub-Saharan Africa remains the country with the highest child mortality rate in the world [1–3]. Ethiopia has one of the highest rates of child deaths and disabilities in the world. More than 704 children die every day from easily preventable diseases [4, 5].

Vaccination is one of the safest and cost-effective interventions to reduce childhood morbidity and mortality [6]. Vaccines prevent an estimated 2.5 million deaths among children under five every year. In 2019, 19.4 million infants did not receive basic vaccines [4, 7, 8]. Ethiopia is the fifth country in the world with a large number of unvaccinated children. In 2018, more nine thousand children were not vaccinated for the third dose of the pentavalent vaccine, and more than 1.2 million children were not vaccinated with the first dose of measles vaccines [5]. Sustainable improvements in service delivery are needed to protect Ethiopian children from unnecessary suffering and death [9, 10].

Various studies have been conducted to identify the determinants of childhood vaccination in different developing countries including Ethiopia [11–17]. These studies investigated the determinants of vaccination coverage through binary logistic regression analysis. The response variable for the above studies was considered as binary (fully vaccinated and not fully vaccinated); as a result, the binary logistic regression model was employed in all the cases. In the case of binary logistic regression, infants who received one or more vaccines considered not vaccinated to fulfill the requirements of binary logistic regression provides sufficient information for studying the pattern of infants who received one or more vaccines. However, the vaccination status of a child is usually classified as fully vaccinated, partially (incomplete) vaccinated, and not vaccinated by considering natural ordering. To answer this, we apply the ordinal logistic regression model to identify the determinants factors of childhood vaccination.

Furthermore, the application of an ordinary logistic regression analysis approach to analyze data in a hierarchical design (i.e. children nested within communities) undermines the assumptions of independence of regression. This paper used a multi-level logistic regression analysis to resolve these limitations and to further assess the significant impact of individual and community-level variables on Ethiopia [18–20]. Therefore, this study aimed to identify the determinants of vaccination coverage among children aged 12–23 months in Ethiopia using multilevel ordinal logistic regression.

## Methods

### Data source and study design

The data for this study was taken from the 2016 EDHS. It is the fourth and most recent nationally representative dataset of demographic and health surveys. The sample was taken using a two-stage stratified sampling. The first stage of the selection was 645 PSU with 202 EAs urban and 443 EAs rural areas based on the 2007 Ethiopian Population and Housing Census (PHC) of the Ethiopian Central Statistics Agency (CSA). All women aged 15–49 years who were usually members of the selected households were eligible for the female survey. All men aged 15–59 years who were usually members of the selected households were eligible for the male survey. Children age 12–23 months with missing age of child and outcome variable were excluded from the study. The relevant data (children recode) on vaccination of children 12–23 months of age were extracted from the EDHS 2016.

### Variable of the study

#### Outcome variable

Child vaccination status was the outcome variable and categorized into three: fully vaccinated, partially (incomplete) vaccinated, and not vaccinated. According to the WHO guideline vaccine [5, 21, 22], full vaccinated is defined as a child between 12 to 23 months who received one dose of Bacille Calmette-Guerin (BCG), at least three doses of pentavalent, three doses of oral polio vaccine (OPV), and one dose of measles vaccine. Partially (incomplete) vaccinated is defined as a child 12 to 23 months who had missed at least one of the eight vaccines [20, 23, 24]. Not vaccinated is also defined as a child 12 to 23 months who didn’t receive any vaccine [20, 24].

#### Independent variables

The independent variables were selected based on prior knowledge and published literature [11, 12, 14, 25–28]. These variables include sex of child, birth order, place of delivery, maternal education, maternal age, number of ANC visits, postnatal care, sex of a child, presence of vaccination card, parental education, parental occupation, household size, wealth index, exposure to mass media, number of living children, mothers tetanus injections received, size of child at birth, ever had terminated pregnancy, wanted last-child, sex of household head, marital status, place of residence, and geographic region.

#### Statistical analysis

R version 3.4.4 statistical software was used to analyze the data. Frequencies and percentages were used to describe the categorical variables. The models for ordinal outcome variables are important in many areas of research since respondents are often classified on an ordinal or graded scale. More importantly, it is often the cases of the respondents are observed nested within clusters or communities (i.e children nested with community/clusters) and so the use of the ordinal regression model which assumes the observations are independent which is problematic. In this case, the multilevel regression model better analyzes the response measurements [29, 30]. A Multilevel Proportional Odds Model (MPOM) was used to identify the individual and community-level factors associated with child vaccination. The proportionality assumptions for MPOM were checked by using Chi-square parallel line tests [29]. MPOM model contains both fixed and random effects. The fixed effect represents the mean response, while the random effect represents the individual level responses. The fixed effect was reported in terms of adjusted proportional odds ratio (APOR) with their 95% CI. All variables with *p*-values < 0.05 have been considered statistically significant. The random effect was also measured by Proportional Change in Variance (PCV) and Intra-class Correlation Coefficient (ICC). Four consecutive models (model I-IV) were observed. The model I (null model) was run to test the inter-group (community) variability on child vaccination and to decide whether the data is fit for multilevel modeling or not. Model II includes only individual-level factors. Model III includes only community-level factors. Model IV includes both individual and community-level factors [19, 20].

#### Model fit statistics

Deviance Information Criteria (DIC) and Akaike’s Information Criterion (AIC) were used to compare and select the model. The model with the minimum value of the information criterion was best fits the data [19, 20].

## Result

### Vaccination coverage in Ethiopia

**Fig. 1:**
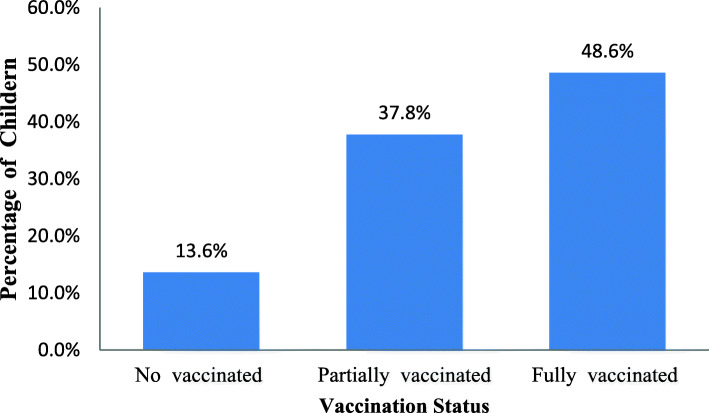
Child vaccination coverage in Ethiopia

**Fig. 2:**
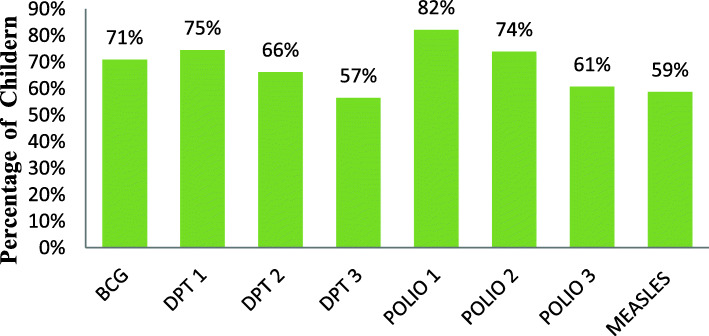
Vaccine specific coverage among children aged 12–23 months in Ethiopia, 2016 EDHS

The full vaccination coverage in Ethiopia was 48.6%. The majority, 71% of the children received BCG. Three fourth percent of the children received both Pentavalent1 and Polio 2. Eighty-two percent the children of received Polio 1. More than half, 59 of them received Measles vaccination. Besides, Vaccine specific coverage for Pentavalent 2, Pentavalent 3, and Polio 2 were 66, 57, and 74%, respectively (Figs. 1 and 2).

### Socio-demographic and economic characteristics of parents and children

A total of 1929women with children 12–23 months of old were included in the study. Regarding parental educational status, 50.5% of fathers and 60.7% of mothers had no formal education; while 30.4% of fathers and 26.3% of mothers attended primary school. More than half percent of mothers (50.7%) were in the age group of 25–34 years. Nearly 60 % of households had a poor wealth index and78.8% of the respondents were living in rural areas. More than three fourth percent of households were headed by males. Almost all (93.4%) mothers are married, and about half (51%) of the children were female. About 47.7%, of mothers, had Muslim beliefs and 35.5% had not ever got antenatal visits (Table 1).

**Table 1:** Socio-demographic and economic characteristics of parents and children aged 12–23 months in Ethiopia, 2016EDHS

Variable	Categories	Frequency	Percentage
Highest educational level	No education	1170	60.7
	Primary	508	26.3
	Secondary and above	251	13.0
Respondent’s current age	15–24	546	28.3
	25–34	978	50.7
	35–49	405	21.0
Number of living children	1–2	786	40.7
	3–4	560	29.0
	5+	583	30.2
Mother occupational status	No	1362	70.6
	Yes	567	29.4
Place of delivery	Home	1101	57.1
	Health facility	828	42.9
Sex of household head	Male	1509	78.2
	Female	420	21.8
Wealth index combined	poor	979	50.8
	Middle	279	14.5
	Rich	671	34.8
Antenatal Care visits	No antenatal visits	690	35.8
	1–3	541	28.0
	4 and above	698	36.2
Husband education level	No education	974	50.5
	Primary	586	30.4
	Secondary and above	369	19.1
Postnatal Care	No	1652	85.6
	Yes	277	14.4
Current marital status	Not Married	128	6.6
	Married	1801	93.4
Sex of child	Male	946	49.0
	Female	983	51.0
Birth order number	First	416	21.6
	2–3	600	31.1
	4 abd above	913	47.3
Presence of vaccination document	No	1080	56.0
	Yes	849	44.0
Wanted last child	Yes	1546	80.1
	No	383	19.9
Relationship to household head	Head	319	16.5
	Wife	1392	72.2
	Others	218	11.3
Religion	Orthodox	607	31.5
	Muslin	921	47.7
	Protestant and others	401	20.8
Size of child at birth	Small	541	28.0
	Average	789	40.9
	Large	599	31.1
Tetanus injections before birth	Not received	809	41.9
	1–2	594	30.8
	3 and above	526	27.3
Ever had a terminated pregnancy	No	1770	91.8
	Yes	159	8.2
Received Vitamin A1	No	929	48.2
	Yes	1000	51.8
Husband/partner’s occupation	No	197	10.2
	Yes	1732	89.8
Type of place of residence	Urban	409	21.2
	Rural	1520	78.8
Region	Tigray	216	11.2
	Afar	171	8.9
	Amhara	178	9.2
	Oromia	287	14.9
	Somali	223	11.6
	Benishangul	156	8.1
	SNNPR	231	12.0
	Gambela	138	7.2
	Harari	117	6.1
	Addis Adaba	102	5.3
	Dire Dawa	110	5.7
Media exposure	No	1265	65.6
	Yes	664	34.4

### **Determinants of** vaccination coverage among children aged 12–23 months

#### Multilevel analysis (fixed-effect analysis)

The results of the multilevel proportional odds model were summarized in Table 2. The score test of proportional odds assumption is found insignificant at 5% level of significance revealed that odds assumption is satisfied. The final selected model (model IV) showed that place of delivery, maternal education, number of ANC visits, presence of vaccination card, maternal occupation, mother and father education, wealth index, mothers tetanus injections received, wanted last-child, place of residence, community-level maternal education, community-level wealth index, community-level media exposure, and geographic region were factors significantly associated with childhood vaccination. Housewife women were 1.522 (AOR =1.522, 95%CI: 1.139, 2.034) times more likely to be partially or fully vaccinated than those employed women. Children who were born at health facilitieswere2.345 (AOR =2.345, 95%CI: 1.766, 3.114) times more likely to be partially or fully vaccinated than those children who were born at home. Children whose mothers made ANC visits at least 4 times during the pregnancy were2.657 (AOR = 2.657; 95% CI: 1.906, 3.704) times more likely to be partially or fully vaccinated than children whose mothers who have not have any antenatal follow up. Compared with non-educated mothers, the odds of partially or fully vaccinated children with mothers who have attended secondary and above education were2.008 times (AOR = 2.008; 95% CI:1.209, 3.334). Likewise, compared to non-educated fathers, the odds of partially or fully vaccinated children for father’s attended primary education were increased by 59.6% (AOR = 1.596; 95% CI: 1.215, 2.096). Children in households in the middle wealth quintile as compared to the poorest had 1.446 (AOR = 1.446; 95% CI: 1.045, 2.002) times more likely to be partially or fully vaccinated. Similarly, children in households in the richer wealth quintile compared to the poorest had 1.679 (AOR = 1.679; 95% CI: 1.233, 2.287) times more likely to be partially or fully vaccinated. The likelihood of being partially or fully vaccinated children among Muslim respondents was0.445 (AOR = 0.445, 95%CI: 0.305, 0.649) times less likely than that for Orthodox Christian respondents. Besides, the probability of being partially or fully vaccinated children among protestant and other respondents was 0.444 (AOR = 0.444, 95%CI: 0.290, 0.678) times less likely than that for Orthodox Christian respondents (Table 2).

**Table 2:** Result of multilevel ordinal logistic regression on child vaccination in Ethiopia, EDHS 2016

Variables	Model I AOR (95% CI)	Model II AOR (95% CI)	Model III AOR (95% CI)	Model IV AOR (95% CI
Place of delivery				
Home		1		1
Health facility		1.497 (1.131, 1.982)^a^		2.345 (1.766, 3.114)^a^
Antenatal Care visits				
No antenatal visits		1		1
1–3		2.188 (1.612, 2.968)^a^		2.069 (1.523,2.810)^a^
4 and above		3.043 (2.198, 4.214)^a^		2.657 (1.906, 3.704)^a^
Presence of vaccination document				
No		1		1
Yes		5.184 (4.011,6.700)^a^		4.590 (3.548, 5.939)^a^
Wanted last child				
No		1		1
Yes		1.390 (1.047, 1.847)^a^		1.436 (1.079, 1.911)^a^
Tetanus injections before birth				
Not received		1		1
1–2		1.307 (0.968, 1.764)		1.361 (1.007, 1.839)^a^
3 and above		1.577 (1.171, 2.122)^a^		1.584 (1.173, 2.137)^a^
Educational status				
No education		1		1
Primary		1.514 (1.138, 2.014)^a^		1.510 (1.134, 2.011)^a^
Secondary and above		2.051 (1.252, 3.359)^a^		2.008 (1.209, 3.334)^a^
Occupational status				
Employed		1		1
Housewife		1.372 (1.062, 1.772)^a^		1.522 (1.139, 2.034)^a^
Wealth index				
Poor		1		1
Middle		1.717 (1.230, 2.397)^a^		1.446 (1.045, 2.002)^a^
Rich		2.085 (1.476, 2.944)^a^		1.679 (1.233, 2.287)^a^
Husband education level				
No education		1		1
Primary		1.369 (1.046, 1.790)^a^		1.596 (1.215, 2.096)^a^
Secondary and above		2.158 (1.782, 2.535)^a^		1.551 (1.053, 2.283)^a^
Religion				
Orthodox		1		1
Muslin		0.549 (0.396, 0.761)^a^		0.445 (0.305, 0.649)^a^
Protestant and others		0.565 (0.388,0.822)^a^		0.444 (0.290, 0.678)^a^
Region				
Tigray			1	1
Afar			0.035 (0.017, 0.075)^a^	0.117 (0.055, 0.250)^a^
Amhara			0.319 (0.155, 0.656)^a^	0.517 (0.279, 0.959)^a^
Oromia			0.111 (0.057, 0.216)^a^	0.234 (0.121, 0.453)^a^
Somali			0.088 (0.043, 0.177)^a^	0.285 (0.136, 0.596)^a^
Benishangul			0.663 (0.307, 1.431)	1.067 (0.525, 2.168)
SNNPR			0.348 (0.176, 0.690)^a^	0.735 (0.369, 1.463)
Gambela			0.096 (0.045, 0.204)^a^	0.170 (0.080, 0.360)^a^
Harari			0.271 (0.118, 0.625)^a^	0.512 (0.225, 1.165)
Addis Adaba			0.943 (0.317, 2.806)	0.779 (0.290, 2.093)
Dire Dawa			1.111 (0.437, 2.826)	1.020 (0.414, 2.512)
Place of residence				
Urban			1	1
Rural			0.378 (0.226, 0.632)^a^	0.505 (0.284, 0.898)^a^
Community level wealth index				
Low			1	1
High			2.636 (1.720, 4.039)^a^	2.158 (1.333, 3.491)^a^
Community level maternal education				
Low			1	1
High			1.957 (1.281, 2.989)^a^	1.814 (1.206, 2.729)^a^
Community level media exposure				
Low			1	1
High			2.108 (1.349, 3.296)^a^	1.758 (1.135, 2.725)^a^

1 = reference category of the categorical variable

^a^Significant at 5% level of significance

A child living in a rural area was 0.505 (AOR=; 95% CI: 0.284, 0.898) times less likely to be partially or fully vaccinated than a child who was urban residents. Children live in Afar (AOR = 0.117; 95% CI: 0.055, 0.250), Amhara (AOR = 0.517; 95% CI: 0.279, 0.959), Oromia (AOR = 2.533; 95% CI: 1.320, 4.861), Somali (AOR = 0.234; 95% CI: 0.121, 0.453), Gambela (AOR = 0.170; 95% CI: 0.080, 0.360) were less likely to be partially or fully vaccinated as compared to children live in Tigray. Children living in communities with a high level of maternal education had increased by 81.4% (AOR = 1.814; 95% CI: 1.206, 2.729) of partially or fully vaccinated as compared to those children living in communities with low maternal education level. Children residing in communities with a high proportion of wealth index had 2.158 (AOR =2.158; 95% CI: 1.333, 3.491) times higher odds of partial or full vaccinated as compared to children residing in communities with a low proportion of wealth index. Furthermore, children living in communities with a high proportion of media exposure were 1.758 (AOR = 1.758; 95% CI: 1.135, 2.725) times more likely to be partially or fully vaccinated as compared to those children living in communities with a low proportion of media exposure (Table 2).

#### Multilevel analysis (random-effects analysis)

The result of random effect analysis has been presented in Table 3. The variation of child vaccination was different across communities. The null model indicated that 55.88% of the total variability for child vaccination was due to differences between clusters, with the remaining unexplained 44.12% which is accounted for by individual differences. The highest (89.73%) PCV in the full model (model IV), revealed that 89.73% of the community-level variation on child vaccination has been explained by the combined factors at both the individual and community levels. The MOR value of child vaccination was 6.95 in the null model; this indicated that there was variation between communities (clustering) since 6.95times higher than the reference (MOR = 1). The unexplained community variation child vaccination was reduced to a MOR of 1.86 when all factors were included in the model. This showed that when all factors are measured, the effects of clustering are still statistically significant in the full models (Table 3).

**Table 3:** Measures of variation and model fit statistics on child vaccination in Ethiopia

Measure of variation	Model I	Model II	Model III	Model IV
Variance (SE)	4.167 (0.531)*	0.979 (0.200)*	1.641 (0.258)*	0.428 (0.173)*
PCV (%)	Reference	76.51	60.62	89.73
ICC (%)	55.88	22.93	33.28	11.51
MOR	6.95	2.56	3.38	1.86
Score test for proportional oddsassumption		X^2^_(10)_ = 7.7, *P-*value = 0.651	X^2^_(12)_ = 21.039, *P-*value = 0.052	X^2^_(26)_ = 35.043, *P-*value = 0.069
**Model fit statistics**				
DIC (−2log likelihood)	3414.892	2891.83	3079.304	**2799.66**
AIC	3420.892	2929.831	3115.305	**2859.66**

*reference *P-*value < 0.0001

## Discussion

This study was conducted to investigate the determinant factors of vaccination coverage among children aged 12–23 months in Ethiopia using the EDHS-2016 dataset. In Ethiopia, the prevalence of full vaccination coverage was 48.6% which indicated that childhood vaccination coverage increased in 2016 compared with 2000, 2005, and 2011 which were 14, 20, and 24% respectively. This can be attributed to the health extension program implementation that increased maternal understanding of the value of child vaccination. This vaccination coverage is lower than 79% in Kenya [31], 51% in Malawi [32], 54% in Uganda [33] and 86.4% in Malaysia [34]. The variations in these coverage rates may be explained by the difference in the data generation processes, the difference between these countries in the coverage of health services including the vaccination program, and the nature of the survey.

The level of parental education positively associated with child vaccination, children born from educated parents were more likely to vaccinate as compared with children born from none-educated counterparts. This supports previous findings where a higher level of maternal and father education is associated with a higher odd of child vaccination [12, 14, 15, 35–38]. The potential reason for this might be the fact that the educated parents may have knowledge of vaccination and child protection and may benefit from full vaccination for their children. The result also showed that children from Orthodox Cristiana religion were more fully immunized compared to other religious groups and this result is consistent with the literature reviewed and contribution from different studies on religion [36, 38]. The attribute of this result needs further investigation.

Children born in a health facility were more likely to vaccinate than those born at home. This is inlined with a finding from previous studies which found that institutional delivery increased the chances of children being fully vaccinated also increased [12, 14, 16, 35, 39–41]. As opposed to those who do not attend health facilities, women who visit health facilities have access to or are exposed to sexual and reproductive health services [40]. Similarly, Increase the number of antenatal visits during pregnancy raises the odds of being fully immunized, a finding that is also confirmed by previous researches [12, 14, 15, 35, 39–43]. A woman provides not only professional care but also advice and education to use postnatal care and vaccination services during institutional delivery. Women who give birth at the health facility often receive details about the schedule and the significance of completing the recommended vaccination [14]. And infants born in a health facility also received the first dose of Hepatitis B within 12 h after birth, BCG, and Polio vaccine when the infant was released [13].

Mothers who received tetanus injections before birth were significantly associated with childhood vaccination. It is learned that increase in the number of received tetanus injections before birth rise the likelihood of childhood to be vaccinated, a finding that is also confirmed by previous researches [27, 43]. This might be because mothers visiting tetanus toxoid vaccination at health facilities during pregnancy may be exposed to knowledge about the benefit of childhood vaccination.

The study also revealed that wealth index was an important variable predicts child vaccination. A higher percentage of vaccination coverage was found among children belonging to a higher wealth index as compared to their counterparts. This confirms previous findings where the odd of childhood vaccination increase with an increase in family income [12, 14, 15, 27, 37, 44].

This could be because children with the lowest family income status have been disadvantage and health services have been difficult to access. Poor families could spend high costs and time to preserve their daily lives. The result also showed that children born from working mothers had a lower odd of vaccination than those of non-working mothers, which confirms with findings from previous studies in Ghana [25], where the working status of the mother is negatively associated with child vaccination.

The current study showed that the presence of vaccination documents was significantly associated with childhood vaccination. Mothers who have shown vaccination documents of their children were more likely to fully vaccinate their children as compared to those who were not able to show their children’s vaccination documents. This supports previous findings where the odds of full vaccination were higher in children born to mothers who had vaccination documents as compared to children born to mothers who had no vaccination documents in Ethiopia [36, 44]. This may be simple to recognize and inform mothers with a vaccination card at a house to house visiting times for health extenuation workers. Mothers with a vaccination document will easily recall the appointment of their child and thus help them complete the vaccination of their child [36]. The type of pregnancy was significantly associated with childhood vaccination. Wanted pregnancies were three times more likely to be vaccinated when compared to unwanted pregnancies. The result is in line with a previous study done in Debre Markos, Ethiopia [42].

Children living in urban areas have been reported to have better vaccination status compared with their rural areas. This finding supports studies in Ethiopia [12] and Afghanistan [11], total vaccination level in urban residence was always higher than in rural residence. This might be due to the fact that urban areas have better access to health facilities and health care practitioners than rural areas. Besides, geographical regions were statistically associated with childhood vaccination. Children living in Afar, Amhara, Oromia, Somali, Gambela were less likely to be vaccinated as compared to children living in Tigray. The result is consistent with a previous study done in Ethiopia [36]. The potential explanation for this regional variation is that there is a discrepancy between regions in the coverage of health services including the vaccination program.

Community-level maternal education, media exposures and wealth index was a strong predictor of child vaccination. Higher community-level maternal education showed a higher odd of child vaccination. Besides, increased media exposure in the community might help to increase the odds of child vaccination in that community. Furthermore, a high concentration of wealth index in a community positively influences child vaccination in that community. Children residing in communities with a high proportion of wealth index had higher odds vaccinated as compared to children residing in communities with a low proportion of wealth index. This is in line with other studies conducted in Ethiopia [36] and the Democratic Republic of Congo [14]. There are poorer health facilities in economically poorer communities; even the distance to the health facilities would be far away. Poorer areas will not invest in education for women, and empowerment for women will be lower [36].

## Conclusion

In this study the coverage of child vaccination was low. Place of delivery, maternal education, number of ANC visits, presence of vaccination card, maternal occupation, mother and father education, wealth index, exposure to mass media, mothers tetanus injections received, wanted last-child, place of residence, community-level maternal education, community-level wealth index, community-level media exposure, and geographic region were factors significantly associated with childhood vaccination. Therefore, there is a need to increase child vaccination coverage by promoting institutional delivery and prenatal care visits, as well as maternal tetanus immunization. Besides, public initiatives needed to improve child vaccination coverage, women’s and husband’s education, poor women, and further advancement of health care services for poor women, housewife women, women living in remote areas should be made to maintain further improvements in child vaccination. Furthermore, policies and programs aimed at addressing cluster variations in child vaccination need to be formulated and their implementation must be strongly pursued.

## Data Availability

The survey datasets used in this study was based on a publicly availabledataset that is freely available online with no participant’s identity fromhttp://www.dhsprogram.com/data/available-datasets.cfm.Approval was sought from MEASURE DHS/ICF International and permissionwas granted for this use.

## Abbreviations

AIC: Akaike’s information criterion
AOR: Adjusted odds ratio
CI: Confidence intervals
DIC: Deviance information criterion
EAs: Enumeration areas
EDHS: Ethiopian demographic and health survey
FP: Family Planning
ICC: Intra-clustercorrelation
MOR: Median odds ratio
PCV: Proportional change in variance
SNNPR: SouthernNations, Nationalities, and People Region
